# Sleep Quality among Breast and Prostate Cancer Patients: A Comparison between Subjective and Objective Measurements

**DOI:** 10.3390/healthcare9070785

**Published:** 2021-06-22

**Authors:** Diana Barsasella, Shabbir Syed-Abdul, Shwetambara Malwade, Terry B. J. Kuo, Ming-Jen Chien, Francisco J. Núñez-Benjumea, Gi-Ming Lai, Ruey-Ho Kao, Hung-Jen Shih, Yu-Ching Wen, Yu-Chuan (Jack) Li, Iván Palomares Carrascosa, Kuan-Jen Bai, Youri C. B. Broekhuizen, Monique W. M. Jaspers

**Affiliations:** 1 Graduate Institute of Biomedical Informatics, College of Medical Science and Technology, Taipei Medical University, Taipei 106, Taiwan; diana.barsasella5@gmail.com (D.B.); drshabbir@tmu.edu.tw (S.S.-A.); jack@tmu.edu.tw (Y.-C.L.); 2 International Center for Health Information Technology (ICHIT), College of Medical Science and Technology, Taipei Medical University, Taipei 106, Taiwan; sv14.kekade@gmail.com (S.M.); youribroekhuizen@me.com (Y.C.B.B.); m.w.jaspers@amc.uva.nl (M.W.M.J.); 3 Health Polytechnic of Health Ministry Tasikmalaya, Tasikmalaya 46115, West Java, Indonesia; 4 School of Gerontology Health Management, College of Nursing, Taipei Medical University, Taipei 106, Taiwan; 5 Institute of Brain Science, Yang-Ming University, Taipei 112, Taiwan; tbjkuo@ym.edu.tw (T.B.J.K.); cmjalbert00920@gmail.com (M.-J.C.); 6 Salumedia Labs, Research Division Adhera Health, Inc., Palo Alto, CA 94304, USA; kikonunez@salumedia.com; 7 Taipei Cancer Center, Taipei Municipal Wanfang Hospital, Taipei Medical University, Taipei 116, Taiwan; gminlai@nhri.org.tw (G.-M.L.); rueykao@tmu.edu.tw (R.-H.K.); 8 Department of Urology, Taipei Municipal Wanfang Hospital, School of Medicine, College of Medicine, Taipei Medical University, Taipei 116, Taiwan; jasta1206@gmail.com (H.-J.S.); s811007@yahoo.com.tw (Y.-C.W.); 9 TMU Research Center of Cancer Translational Medicine, Taipei Medical University, Taipei 106, Taiwan; 10 Department of Computer Science and Information Engineering, National Cheng Kung University, Tainan 70101, Taiwan; ivanpc@ugr.es; 11 Andalusian Research Institute of Data Science and Computational Intelligence (DaSCI), University of Granada, 18071 Granada, Spain; 12 Division of Pulmonary Medicine, Department of Internal Medicine, Wanfang Hospital, Taipei Medical University, Taipei 116, Taiwan; 13 Center of Human Factors Engineering of Health Information Technology (HIT-Lab), Department of Medical Informatics, Amsterdam Public Health Research Institute—Location Academic Medical Center (AMC), University of Amsterdam (UvA), 1012 WX Amsterdam, The Netherlands

**Keywords:** breast cancer, prostate cancer, sleep quality, PSQI, actigraphy device, wearable sensors, medical aid systems, medical data analysis

## Abstract

Breast and prostate cancer patients may experience physical and psychological distress, and a possible decrease in sleep quality. Subjective and objective methods measure different aspects of sleep quality. Our study attempted to determine differences between objective and subjective measurements of sleep quality using bivariate and Pearson’s correlation data analysis. Forty breast (*n* = 20) and prostate (*n* = 20) cancer patients were recruited in this observational study. Participants were given an actigraphy device (ACT) and asked to continuously wear it for seven consecutive days, for objective data collection. Following this period, they filled out the Pittsburgh Sleep Quality Index Questionnaire (PSQI) to collect subjective data on sleep quality. The correlation results showed that, for breast cancer patients, PSQI sleep duration was moderately correlated with ACT total sleeping time (TST) (r = −0.534, *p* < 0.05), and PSQI daytime dysfunction was related to ACT efficiency (r = 0.521, *p* < 0.05). For prostate cancer patients, PSQI sleep disturbances were related to ACT TST (r = 0.626, *p* < 0.05). Both objective and subjective measurements are important in validating and determining details of sleep quality, with combined results being more insightful, and can also help in personalized care to further improve quality of life among cancer patients.

## 1. Introduction

Breast and prostate cancers are among the first ten most common forms of cancer in Taiwan, with nearly 14,000 and 6000 cases, respectively, in 2017, and since then these numbers have been growing [1]. Due to technological and medical advancements in the early detection and treatment of breast and prostate cancers, the reported number of cancer patients has increased [2]. This trend has not only occurred in Taiwan, but also in other parts of the world as well. The International Agency for Research on Cancer estimates the global prevalence to be roughly 1.67 million and 1.1 million for breast cancer and prostate cancer, respectively [3]. Moreover, both these cancer types have more favorable survival rates; however, this also depends upon the development level in any given region and the availability of treatment facilities, stage of diagnosis, age, etc. In 2015, breast cancer was the most common cancer in women worldwide [4], whereas prostate cancer was reported as the most common cancer in men [5].

Large numbers of breast and prostate cancer patients experience both physical and psychological side effects, even years after treatment [6,7,8]. Sleep disturbance is one of the major problems in cancer patients, with incidence rates more than 30% [9], which are higher than in the general population [10]. Cancer and cancer treatments are among the contributing factors towards sleep disturbances [11,12]. Sleep quality is one of the major aspects that influences the quality of life (QoL) of these patients [13,14,15]. Sleep problems can also lead to poor healing, increased chances of cancer recurrence, reduced work productivity, poor relationships, and increased use of medications and treatments, which in turn results in increased healthcare costs [16]. The proportion of sleep disturbances among breast cancer survivors was found to be higher than that observed in healthy women [17]. Fortner et al. studied sleep quality among breast cancer patients (*n* = 72) using the Pittsburgh Sleep Quality Index (PSQI) questionnaire. Their results showed that 42% of participants indicated that they had used medication for sleep in the past month, and 21% for the past 3 months [18]. Costa et al. also investigated sleep disturbances in women with breast cancer, and their systematic review reported that breast cancer patients generally reported higher levels of sleep disturbances after treatment compared to before treatment [12]. Both studies, among others, indicated that more-robust evidence is needed to fully support the statements made and concluded that more research into this topic is required to enhance the quality of these results [12,18]. Prostate cancer patients receiving adjuvant therapy have also reported insomnia and sleep disturbances [19,20]. Mitteldorf et al. studied sleep quality among prostate cancer patients (*n =* 973) using the PSQI, which indicated that 75.90% of the total participants suffered sleep distress [21].

Current data analysis methods for examining sleep quality can be divided into two categories: objective (such as actigraphy, polysomnography, etc.) and subjective (such as the Pittsburgh Sleep Quality Index, the Epworth Sleepiness Scale, etc.) [10]. The difference between the two outcomes in a given patient can be defined as that patient’s self-awareness. Objective measurements can better differentiate between sleep and wake [22], whereas subjective ones can determine the effects of the sleep disturbance on a patient’s life [23]. Subjective methods are easy to use, convenient, less expensive, and they reflect personal experience. However, they are prone to reporting bias and are liable to missing or inaccurate data when participants fail to complete them in a timely manner [24]. Their subjectivity, and a tendency to pose a burden in the case of frequent use, could be another disadvantage of subjective methods [25]. Objective methods, on the other hand, can collect detailed data without having the patient frequently report his/her sleep information. However, data management, analysis, and interpretation can be time-consuming [25]. In cases where sleep quality is more vital to a person’s health compared to a healthy population, such as in breast and prostate cancer patients, it is important to understand how these two aspects of sleep quality relate to each other.

Therefore, our study attempted to understand and compare sleep quality between objective and subjective measurements, respectively collected through actigraphy devices (ACTs) and a sleep quality questionnaire, for breast and prostate cancer patients. Correlation analysis have been widely used to obtain an understanding between the two main sources of measurements. Comparing the similarities and differences between the two methods could help to overcome the limitations of utilizing only one method to determine sleep quality. Moreover, determining more specific sleep problems can lead to improved medical aid systems and better treatments and disease management for cancer patients.

### Related Work

Although sleep quality has been measured since the 1980s, there have been increasing interests in applying subjective and objective measurements, as per a review by Landry et al. [26]. While the PSQI is a widely used measure to assess subjective sleep quality, the Consensus Sleep Diary is also being used for insomnia research and application for poor and good sleepers [27]. Polysomnography has been considered as the gold standard objective measure of sleep. It is said to provide the most accurate assessment of sleep quality and quantity measures [28]. However, it requires an overnight stay in a sleep laboratory or clinic, which limits continuous measurements for long periods of time. A wrist-worn actigraphy device is another widely used tool for objective sleep quality. The devices are battery powered, light-weight, non-invasive, and contain accelerometers measuring tri-axial movement. They are more practical for long term measurements at home [28,29]. ACT and PSG measurements were assessed by Kanady et al. among healthy individuals, using Bland–Altman analyses [30], whereas Marino et al. compared the measurements among older adults using Spearman rank correlation [31]. Grandner et al. first correlated the PSQI and ACTs in a non-clinical sample of young and older adults [32]. Grutsch et al. investigated the relationship of daily activity/sleep time for ACT and PSQI measures among lung cancer patients using analysis of autocorrelation [33]. Another study comparing ACT and PSQI measures among breast cancer patients showed difference in measurements using the Bland and Altman limits of agreement method [34]. All the studies showed some correlations between the subjective and objective measures, as conducted using different measures and methods of analysis. The novelty of our study is that it compares subjective and objective sleep quality measures for breast and prostate cancer patients using the PSQI and ACTs. The correlation is described using bivariate and Pearson’s correlation analysis.

## 2. Materials and Methods

In total, 40 participants (20 breast cancer patients and 20 prostate cancer patients) were recruited in April, May, and June, 2018 from two cancer centers in Taipei. A study nurse approached the patients, explained the study aims, and further recruited them after they signed a consent form. Patients were included if (1) they were 20 years or older; (2) had been diagnosed with either breast or prostate cancer; (3) were receiving evaluation, treatment, or follow-up care at Wan Fang Hospital or Taipei Medical University Hospital at the time of enrollment; (4) able to understand Mandarin Chinese; (5) able to give informed consent to participate in the study. Patients were excluded if (1) they could not understand the intent of the study or (2) the treating clinician believed that the patient was not fit to participate. To achieve 80% statistical power at a 5% significance level, and an effect size of 1.1, a sample of at least 30 patients would be needed. Foreseeing withdrawals and missing data, the number of participants was set to permit a loss of up to 25% of patients.

The actigraphy devices (Actigraphy device model no.: XB40ACT; engineered in-house by K&Y Labs, Taipei, Taiwan) included in this study were manufactured in Taiwan and had been used in other institutional review board (IRB)-approved studies at Yang-Ming University Hospital and Taipei City Hospital. The sensor is a small device with dimensions of 44 × 19 × 8 mm ^3^, weighing about 7 g. It consists of an 80-mAh lithium ion battery that works for up to 14 days. The sensor is worn by the user and collects data that are transmitted via Bluetooth to a mobile device. These data are then transferred and stored in the cloud, from where they can be downloaded for analysis. The sensor was validated by Kuo et al. [35]. This device collects three-dimensional data every second and converts those data into 10-s movement statistics. These measurements include milligravity and differences in angle and spin. Each sensor has a unique device ID that allows for movement data to be linked to other collected variables.

In this cohort study, data were prospectively collected. Ethical approval for the study was obtained from the Taipei Medical University-Joint Institutional Review Board under the committee approval number N201803041. After a patient was deemed eligible for the study and had signed the consent form, he or she received the actigraphy device, with instructions to wear it continuously during day and night times for a duration of 7 days. At the end of the study period, participants returned the actigraphy device and filled in the Chinese version of the PSQI questionnaire.

### 2.1. Measuring Sleep Quality Levels

The objective sleep data were measured using the actigraphy device. Such devices monitor continuous movements and have been used to determine disruptions in sleep wake cycles [36,37] and been validated among cancer patients [33]. The sleep/wake detection method used was based on an algorithm presented by Gorny et al. [38]. This algorithm splits the sleep data in blocks of 30 s each, called ‘epochs’. The formula is as follows:

(*A_act_ + B_act_ + E_act_*), (1)

where a critical value, K, is maintained. If (*A_act_* + *B_act_* + *E_act_*) ≥ K, the epoch is scored as awake, whereas if (*A_act_* + *B_act_* + *E_act_*) < K, the epoch is labeled as asleep. In this formula, *A_act_* stands for the overall activity in the last four epochs, *B_act_* is the overall activity in the next four epochs, and *E_act_* is the overall activity in the current epoch.

The subjective sleep data were quantified through the PSQI questionnaire. The PSQI measures seven subscales (*sc*_1_, sleep quality; *sc*_2_, latency; *sc*_3_, duration; *sc*_4_, efficiency; *sc*_5_, disturbance; *sc_6_*, medication use; and *sc*_7_, dysfunction). The scores for each of these subscales range from 0 (no difficulty) to 3 (severe difficulty) and are combined into a single sleep quality score, denoted *PSQI*, as follows:

(2) $$PSQI=\sum_{k=1}^{7}sc_{k}$$

where *PSQI* ∈ [0, 21].

Minimum Score = 0 (better); Maximum Score = 21 (worse).
Interpretation: PSQI < 5 associated with good sleep quality.
PSQI ≥ 5 associated with poor sleep quality.

i.e., the resulting score is scaled 0~21, where *PSQI* = 0 represents perfect sleep health and *PSQI = 21* indicates severe difficulties in this area. The Chinese version of the *PSQI* was validated [39] and has extensively been used in clinical and research studies to measure sleep quality [40,41,42].

#### 2.1.1. Data Analysis

The actigraphy device (abbreviated as ACT from now onward) collected the activity data of four variables: activity level in milligravity (mg), angle, spin (both in radians), and impact. These variables were stored every 10 s.

These movement data were split into four variables. The total amount of time that the patient was asleep was named the total sleeping time (TST). The ‘sleep efficiency’, denoted SE ∈ [0, 1], is the TST divided by the total time spent in bed:

(3) $$\mathrm{SE}=\frac{\mathrm{TST}}{Total\;time\;spent\;in\;bed}$$

The wake after sleep onset (WASO) refers to the duration of periods in which the patient was awake after he/she went to sleep, where the total amount of awakenings was recorded under the number of awakenings (NAW). The duration of the period between the patient going to bed and the patient falling asleep was recorded as the sleep onset latency (SOL).

**Figure 1:**
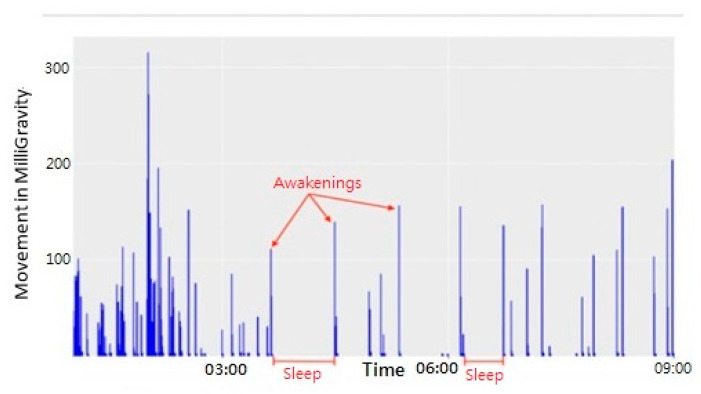
Movement data during sleep.

Figure 1 shows how the movement data were used to analyze sleep. Spikes in the data indicate when the participant was active (most likely turning over in bed), while the empty times in between the spikes indicate that the participant was asleep. The NAW is also visible in this figure; all spikes that exceed a value of 100 were counted as awakenings [43].

The raw movement data were analyzed using proprietary software developed in-house.

**Figure 2:**
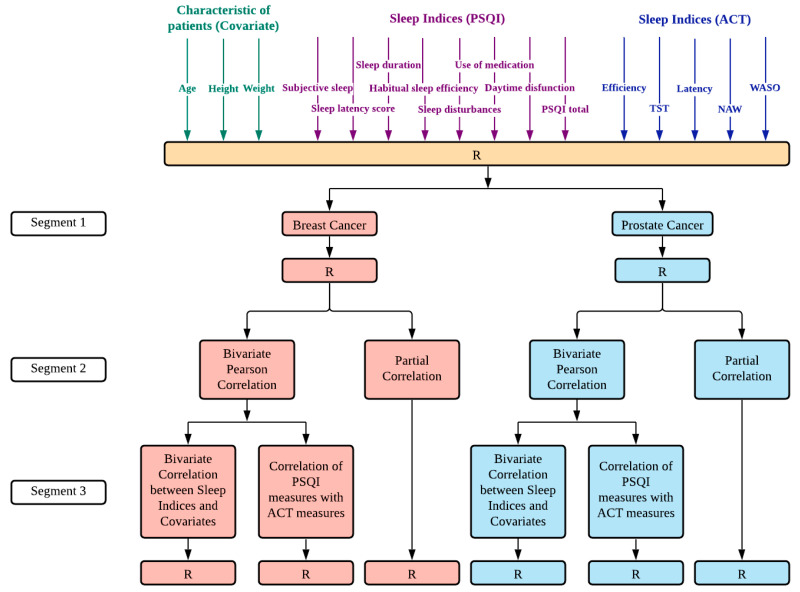
Step-by-step pipeline from data collection to obtaining results for correlation analysis. Segment 1: Dataset consisted of variables that included the characteristics of patients, sleep indices measured by PSQI, and sleep indices measured by actigraphy. The collected data was divided into breast cancer and prostate cancer datasets. Segment 2: We conducted bivariate Pearson correlation and partial correlation for each dataset. Segment 3: Bivariate Pearson correlation analysis resulted in the bivariate correlation between sleep indices and covariates, and correlation of PSQI measures with actigraphy measures. R—Result, after variables selection or statistical analysis.

The variables were analyzed and compared using SPSS statistics software (version 23.0.0.0, International Business Machines Corporation (IBM), New York, United States of America). The data analysis was divided into two sections (Figure 2).

First, necessary variables to develop insights into the participant’s sleep data were calculated from the raw accelerometer measurements. These values were entered into a dataset in SPSS, together with the outcomes of the PSQI questionnaire. After the data were verified, we conducted the bivariate correlation between sleep indices and covariates (age, weight, and height) in breast cancer and prostate cancer patients. We ran a Pearson correlation between the objective and subjective scores to verify whether a significant correlation was present.

Further, partial correlations were carried out to determine if any observed associations among the sleep quality indices could be attributed to individual differences in age, height, or weight.

We also conducted cross-tabulation and one way ANOVA for ACT-sleep quality and PSQI-sleep quality among breast and prostate cancer patients; *p* values < 0.05 were considered statistically significant, while *p* values < 0.01 indicated strong statistical significance. Missing data were managed by applying the mean substitution method. The average value of a variable is used instead of the missing data value for the same variable. This allows researchers to utilize data collected in incomplete datasets [44] All participants were given a unique case ID. By storing the data under the unique case ID rather than using personal information, the collected data were anonymized from the beginning. During the research period, all members of the research team were allowed to access the (anonymized) research data.

#### 2.1.2. Comparison of Good vs. Poor Sleep Quality

The sleep quality was determined based on the sleep efficiency, SE, which is the percentage of total sleep time divided by total time in bed. It was calculated on a night-by-night basis, and these scores were averaged. Individuals were classified as having (1) good sleep efficiency based on *S*E ≥ 85, (2) poor sleep efficiency based on *S*E ≤ 75, or (3) average sleep efficiency based on *S*E < 85 and *S*E > 75 [26]. A *PSQI* total score of >5 is indicative of poor sleep [45].

## 3. Results

### 3.1. Descriptive Characteristics

Although 40 patients were recruited, only the data of 31 patients were usable due to a technical malfunction in nine of the actigraphy devices. The participants that used these malfunctioning devices were excluded from the data analysis altogether. All the remaining 31 participants completed the study. Thus, 16 breast cancer patients and 15 prostate cancer patients were included in the study.

The baseline demographic characteristics and the measured sleep variables of our sample of participants are shown in Table 1.

### 3.2. Bivariate Correlation Analysis between the Sleep Measures and the Covariates of Interest

The correlation between various sleep indices and covariates are described in Table 2. Pearson product-moment correlations (r) were calculated for all continuous variables.

The sleep indices were unrelated to the covariates for both the cancer types, as there was no indicated statistical significance.

### 3.3. Correlations between the Subjective and Objective Sleep Measures

Correlations determining associations between PSQI measures and ACT measures among the patients are shown in Table 3.

For breast cancer patients, PSQI sleep duration was moderately associated with ACT TST (*r* = −0.534, *p* < 0.05), and PSQI daytime dysfunction was related with ACT efficiency (*r* = 0.521, *p* < 0.05). For prostate cancer patients, PSQI sleep disturbances were related to ACT TST (*r* = 0.626, *p* < 0.05). The results of correlation were almost similar between bivariate and partial correlations. This implied that the covariates did not change the correlation values.

### 3.4. Cross Tabulation for Sleep Quality Measures

Cross-tabulation between ACT and PSQI are shown in Table 4. The chi-square test showed significance (*p* = 0.027) for breast cancer, whereas there was no significance for prostate cancer.

Further, as shown in Table 5, patients were classified as those who underestimate on the PSQI (PSQI indicated sleep quality was poor but ACT indicated it was average or good), Accurate (PSQI indicated sleep quality category matched with the ACT indicated one) or overestimate (PSQI indicated sleep quality was good but ACT indicated that it was relatively poor). In the case of breast cancer, the maximum number of patients were classified under PSQI over-estimators (*n* = 10), whereas, among prostate cancer patients, most of them were accurate on the PSQI (*n* = 8).

## 4. Discussion

The aim of this research was to determine similarities and differences between self-reported subjective sleep quality and objective sleep quality, as perceived by an actigraphy device. All 40 participants finished the study, but data from only 31 participants were analyzed, due to technical issues. Our results show that breast cancer patients had an average total PSQI score close to 5 and prostate cancer patients had an average total PSQI score of >5, which is considered poor sleep quality.

The discrepancies between PSQI and ACT observed sleep quality are explained in Table 5. Surprisingly, for breast cancer, most patients (62.5%) were over-estimators (patients whose PSQI defined sleep quality was good or average but their ACT-defined sleep quality was poor), whereas, for prostate cancer patients, 53.33% were in general accurate estimators in subjective terms (patients whose PSQI defined sleep quality matched the ACT determined sleep quality). However, since there were no significant differences in these groups, the demographic variables do not explain the discrepancy between the PSQI and ACT measured sleep quality.

Some of the variables showed significant correlations (Table 3), which were unaffected by demographic variables, implying that the correlations are likely to be true associations. Moreover, there were some significant correlations between some of the PSQI and ACT measures. For breast cancer, the ACT efficiency measures were correlated to PSQI measures for daytime function; ACT TST were correlated to PSQI sleep duration. These findings were consistent with previous studies conducted among breast cancer patients. Fontes et al. reported a correlation between the TST and sleep duration among breast cancer patients, who used the ACT device for five consecutive days [46]. Similar findings were indicated by Jakobson et al., who examined the sleep quality in hospitalized patients with advanced cancers [47]. For prostate cancer, ACT TST were correlated to PSQI measures for sleep disturbances. Despite these correlations found in our study, we did not find any significant findings in the PSQI and ACT sleep latency measures, which were similar to findings in a previous study [46]. Our findings indicated moderate to low correlations between the ACT and PSQI measures, as also observed in previous oncological treatment studies for subjective and objective measurements [48,49]. The use of medications was not associated with any of the ACT measures for both the cancer patients in our study.

Sleep efficiency and sleep disturbance, which might be difficult for patients to monitor, were not significantly correlated. This is in line with related work, such as studies by Grandner et al. and Landry et al., conducted among adults over 55 years of age [26,32]. Similar to our results, they found no correlation between objective and subjective measurements of variables such as sleep efficiency and concluded that subjective measurements do not provide predictive validity for objective sleep quality. However, sleep efficiency indicates whether a patient is getting enough hours of sleep, which means that it is an important parameter to be assessed in sleep studies.

Overall, our results suggest that subjective measures provide a different aspect of sleep quality when compared to objective measures. A review study by Madsen et al. also indicated that subjective and objective sleep measures may not necessarily illustrate the same sleep dimension, but may illustrate various effects of the sleep elements expressing sleep disturbances [50]. By comparing the retrieved objective data to the subjective outcomes of the questionnaire, our aim was to better understand how actigraphy devices can be applied to measure and track the sleep quality of breast and prostate cancer patients. Our results indicated that while the actigraphy device produced a reliable representation of ‘quantitative’ variables such as TST and sleep latency, it is still recommended to collect both objective and subjective scores for an accurate sleep profile of breast and prostate cancer patients. PSQI scores provide efficient measures for subjective sleep quality among cancer patients [51], and they definitely represent an important aspect. Actigraphy devices are also seen to be effective in the evaluation of sleep quality [33,52]. The evaluation efficiency increased when combined with subjective measurements [37,53].

Therefore, this study provides evidence that both objective and subjective measurements are important in validating and determining the details of sleep quality for patients with breast and prostate cancer. This may lead to better definitions of patients’ sleep profiles and improve the existing medical aid systems, namely in terms of enhanced personalized support and care provided for their sleep and QoL following cancer treatment. Not only will the support and treatment be palliative but they will also aid in determining preventive measures for those undergoing similar cancer treatments. Additionally, the use of objective measurements, through monitoring sensors, can aid physicians in detecting possible major sleep disorders among breast and prostate cancer patients. As a part of a future study, we aim to correlate daytime activity and sleep with the QoL of cancer patients.

One of this study’s limitations relates to wearable actigraphy not being as accurate as the golden standard for objective sleep measurements: polysomnography. Results from the study by Dean et al. [54] suggested that actigraphy might overestimate the total sleep time in patients with insomnia. Because actigraphy requires physical movement, a participant might have been awake but not moving, causing the actigraphy device to detect sleep. However, polysomnography is costly and invasive, and therefore it was not feasible to use that method in this study.

Furthermore, a small sample size increased the possibility of more missing data considering technical issues. Using the device for a longer duration would further facilitate efficient recording of the data for different sleep patterns over different time periods. Finally, the course of the disease or specifications of the stages for patients recruited in the study could have been interesting for additional insights in the covariate analysis. Inclusion of such information could be considered in future studies.

## 5. Conclusions

Combined measurements as obtained from actigraphy devices as well as questionnaires help in determining actual sleep parameters that are affecting sleep quality, and further help in providing personalized care to cancer patients to improve their QoL. We expect that a good sleep quality will contribute to a better QoL for Taiwanese breast and prostate cancer patients. It might also cover the bases for possible intervention studies in the future about sleep quality with breast or prostate cancer patients.

## Figures and Tables

**Table 1:** Descriptive statistics for the study variables in breast and prostate cancer.

	Breast Cancer (*n* = 16)		Prostate Cancer (*n* = 15)	
Variable	Mean (SD)	Range	Mean (SD)	Range
Age (years)	60.0000 (8.79)	45.00–76.00	75.13(12.65)	59.00–99.00
Height (cm)	156.2125 (5.26)	148.20–163.00	165.23 (8.43)	141.00–180.00
Weight (kg)	60.17 (9.83)	49.00–85.00	70.84 (6.39)	59.00–80.00
(PSQI) What time to bed (time in hh:mm)	21:54 (5:10)	20:00–2:30	22:24 (1:12)	20:00–24:00
(PSQI) What time out of bed (time in h:mm)	6:58 (1:05)	5:00–8:30	6:09 (0:53)	4:30–8:00
(PSQI) Subjective sleep quality (score, 0–3)	1.13 (0.72)	0–3	1 (0.854)	0–2
(PSQI) Sleep latency score (score, 0–3)	0.73 (1.03)	0–3	1.00 (0.85)	0–2
(PSQI) Sleep duration (score, 0–3)	0.56 (0.63)	0–2	1.13 (1.19)	0–3
(PSQI) Habitual sleep efficiency (score, 0–3)	0.13 (0.34)	0–1	0.60 (0.91)	0–3
(PSQI) Sleep disturbances (score, 0–3)	1.38 (0.50)	1–2	0.40 (0.63)	0–2
(PSQI) Use of medication (score, 0–3)	0.31 (0.79)	0–3	1.60 (0.63)	1–3
(PSQI) Daytime dysfunction (score, 0–3)	0.38 (0.81)	0–3	0.93 (1.22)	0–3
(PSQI) Total hours of sleep (time in h:mm)	7:33 (1:29)	5:00–10:30	7:14 (1:29)	4:00–10:00
PSQI Total (score, 0–21)	4.88 (2.28)	2–9	5.93 (1.62)	4–10
ACT efficiency (score, 0–100)	71.4475 (9.23)	55.82–85.90	62.26 (12.32)	41.57–84.94
ACT TST (time in h:mm:ss)	5:46:33 (1:20:30)	3:17:21–7:35:22	4:50:13 (1:14:37)	2:43:20–6:49:54
ACT Latency (time in h:mm:ss)	0:32:21 (0:26:06)	0:03:44–1:44:18	0:30:15 (0:26:51)	0:02:05–1:50:32
ACT NAW (number of awakenings)	12.80 (4.34)	6.60–20.86	13.06(3.30)	7.86–20.43
ACT WASO (time in h:mm:ss)	1:52:22 (0:49:40)	0:51:16–3:17:16	2:07:37 (0:36:45)	1:05:41–3:35:25

**Table 2:** Bivariate correlation between sleep indices and covariates in breast cancer and prostate cancer patients.

	Breast Cancer			Prostate Cancer		
Sleep Indices	Covariates			Covariates		
	Age	Height	Weight	Age	Height	Weight
Subjective sleep quality (PSQI)	−0.042	−0.145	−0.232	0.087	0.185	−0.088
Sleep latency score (PSQI)	−0.319	−0.357	−0.475	0.251	0.070	0.235
Sleep duration (PSQI)	0.193	−0.004	−0.148	−0.231	0.007	0.116
Habitual sleep efficiency (PSQI)	0.333	0.121	−0.170	−0.382	0.033	0.359
Sleep disturbances (PSQI)	0.091	0.366	0.119	0.096	−0.020	0.024
Use of medication (PSQI)	0.105	−0.384	−0.178	0.250	0.192	0.011
Daytime dysfunction (PSQI)	−0.122	0.310	0.336	0.154	0.057	−0.497
PSQI Total	−0.070	−0.319	−0.416	0.219	0.318	0.209
ACT efficiency	−0.238	0.062	−0.059	0.060	−0.161	−0.270
ACT TST	−0.180	0.107	−0.177	0.099	−0.142	−0.043
ACT Latency	0.077	0.064	0.025	−0.001	0.193	0.182
ACT NAW	0.079	0.020	−0.032	0.229	−0.212	−0.254
ACT WASO	0.223	0.000	−0.055	−0.127	0.210	0.308

**Table 3:** Correlations of PSQI measures with ACT measures in breast cancer and prostate cancer.

	PSQI Measures—Breast Cancer							
ACT Measures	Subjective Sleep Quality	Sleep Latency Score	Sleep Duration	Habitual Sleep Efficiency	Sleep Disturbances	Use of Medication	Daytime Dysfunction	PSQI Total
ACT efficiency	0.281/0.254	0.062/−0.128	0.230/0.366	0.156/0.261	0.108/0.012	−0.263/−0.320	0.521 */0.654 *	0.480/0.504
ACT TST	0.311/0.261	0.308/0.248	−0.534 */−0.604 *	0.444/0.502	0.149/0.016	−0.021/−0.033	−0.017/0.049	0.196/0.084
ACT Latency	0.100/0.190	0.254/0.523	−0.225/−0.322	−0.046/−0.054	−0.196/−0.149	0.110/0.195	−0.477/−0.645 *	−0.198/−0.097
ACT NAW	−0.134/−0.188	−0.018/−0.039	−0.388/−0.457	0.259/0.195	0.081/−0.017	−0.098/−0.137	−0.251/−0.227	−0.299/−0.453
ACT WASO	−0.158/−0.187	0.014/0.132	−0.487/−0.663 *	0.225/0.084	0.195/0.198	0.361/0.392	−0.287/−0.258	−0.209//−0288
**ACT measures**	**PSQI Measures—Prostate Cancer**							
ACT efficiency	−0.294/−0.318	−0.172/−0.105	−0.152/−0.144	−0.045/0.037	0.442/0.474	0.356/0.440	−0.116/−0.286	0.026/0.148
ACT TST	−0.105/−0.090	0.015/0.003	−0.501/−0.509	−0.109/−0.099	0.626 */0.628 *	0.304/0.340	−0.001/0.006	0.105/0.149
ACT Latency	0.414/0.415	0.097/0.024	−0.022/−0.020	−0.163/−0.221	−0.212/−0.231	−0.449/−0.568	0.384/0.541	−0.102/−0.255
ACT NAW	−0.087/−0.101	−0.071/−0.066	−0.228/−0.190	−0.104/0.017	−0.321/−0.353	0.211/0.229	0.044/−0.071	−0.219/−0.214
ACT WASO	0.023/0.026	0.061/−0.012	−0.071/−0.112	0.194/−0.112	−0.223/−0.241	−0.014/−0.047	0.080/0.260	0.018/−0.095

Pearson Correlations appear on the left side of the forward slash and partial correlations appear on the right side. Partial correlations are covaried for participant age, height, and weight. * Correlation is significant at the 0.05 level (2-tailed). Bold, indicate significant correlation.

**Table 4:** Cross Tabulation of ACT-sleep quality vs. PSQI-sleep quality in breast and prostate cancer patients.

		PSQI Sleep Category		
Category		Breast Cancer		
		Good	Poor	Total
ACT	Good	0	1	1
	Poor	8	1	9
	Average	2	4	6
	Total	10	6	16
*p* value	0.027			
X^2^	7.253			
		Prostate Cancer		
ACT	Good	0	0	0
	Poor	4	8	12
	Average	2	1	3
	Total	6	9	15
*p* value	0.693			
X^2^	0.156			

**Table 5:** Comparison of accurate and inaccurate self-reports on the PSQI on demographics in breast cancer and prostate cancer.

		**Breast Cancer**		
Variable	Underestimate on PSQI *n* *= 5*	Accurate on PSQI *n* *= 1*	Overestimate on PSQI *n* *= 10*	*p* value
	Mean (SD) or *n* (%)	Mean (SD) or *n* (%)	Mean (SD) or *n* (%)	
Age	59.60 (8.20366)	56 (-)	60.60 (9.83418)	0.891
Height	156.54 (4.96065)	150 (-)	156.67 (5.53374)	0.507
Weight	55.36 (5.36172)	49 (-)	63.70 (10.38214)	0.152
		**Prostate Cancer**		
	Underestimate on PSQI *n = 1*	Accurate on PSQI *n = 8*	Overestimate on PSQI *n = 6*	*p* value
	Mean (SD) or *n* (%)	Mean (SD) or *n* (%)	Mean (SD) or *n* (%)	
Age	99.00 (-)	75.25 (11.39862)	71.00 (11.48913)	0.118
Height	163.10 (-)	167.88 (2.86294)	162.07 (12.79166)	0.461
Weight	69.90 (-)	71.79 (7.49027)	69.73 (5.74584)	0.849

Italics, number of patients.

## Data Availability

The authors cannot provide the data. The results of this study will be published, and part of the collected data will be made public in the open access repository of the CATCH project. Public datasets will be fully anonymized and protected in accordance with European regulations and laws (2002/58/EC [55] and 95/46/EC [56]), which regulate the privacy of electronic communications and the processing and free movement of personal data.
