# Erratum: Barsasella et al. Sleep Quality among Breast and Prostate Cancer Patients: A Comparison between Subjective and Objective Measurements. *Healthcare* 2021, *9*, 785

**DOI:** 10.3390/healthcare9111581

**Published:** 2021-11-19

**Authors:** Diana Barsasella, Shabbir Syed-Abdul, Shwetambara Malwade, Terry B. J. Kuo, Ming-Jen Chien, Francisco J. Núñez-Benjumea, Gi-Ming Lai, Ruey-Ho Kao, Hung-Jen Shih, Yu-Ching Wen, Yu-Chuan (Jack) Li, Iván Palomares Carrascosa, Kuan-Jen Bai, Youri C. B. Broekhuizen, Monique W. M. Jaspers

**Affiliations:** 1 Graduate Institute of Biomedical Informatics, College of Medical Science and Technology, Taipei Medical University, Taipei 106, Taiwan; diana.barsasella5@gmail.com (D.B.); drshabbir@tmu.edu.tw (S.S.-A.); jack@tmu.edu.tw (Y.-C.L.); 2 International Center for Health Information Technology (ICHIT), College of Medical Science and Technology, Taipei Medical University, Taipei 106, Taiwan; sv14.kekade@gmail.com (S.M.); youribroekhuizen@me.com (Y.C.B.B.); m.w.jaspers@amc.uva.nl (M.W.M.J.); 3 Health Polytechnic of Health Ministry Tasikmalaya, Tasikmalaya 46115, West Java, Indonesia; 4 School of Gerontology Health Management, College of Nursing, Taipei Medical University, Taipei 106, Taiwan; 5 Institute of Brain Science, Yang-Ming University, Taipei 112, Taiwan; tbjkuo@ym.edu.tw (T.B.J.K.); cmjalbert00920@gmail.com (M.-J.C.); 6 Salumedia Labs, Research Division Adhera Health, Inc., Palo Alto, CA 94304, USA; kikonunez@salumedia.com; 7 Taipei Cancer Center, Taipei Municipal Wanfang Hospital, Taipei Medical University, Taipei 116, Taiwan; gminlai@nhri.org.tw (G.-M.L.); rueykao@tmu.edu.tw (R.-H.K.); 8 Department of Urology, Taipei Municipal Wanfang Hospital, School of Medicine, College of Medicine, Taipei Medical University, Taipei 116, Taiwan; jasta1206@gmail.com (H.-J.S.); s811007@yahoo.com.tw (Y.-C.W.); 9 TMU Research Center of Cancer Translational Medicine, Taipei Medical University, Taipei 106, Taiwan; 10 Department of Computer Science and Information Engineering, National Cheng Kung University, Tainan 70101, Taiwan; ivanpc@ugr.es; 11 Andalusian Research Institute of Data Science and Computational Intelligence (DaSCI), University of Granada, 18071 Granada, Spain; 12 Division of Pulmonary Medicine, Department of Internal Medicine, Wanfang Hospital, Taipei Medical University, Taipei 116, Taiwan; 13 Center of Human Factors Engineering of Health Information Technology (HIT-Lab), Department of Medical Informatics, Amsterdam Public Health Research Institute—Location Academic Medical Center (AMC), University of Amsterdam (UvA), 1012 WX Amsterdam, The Netherlands

The authors wish to make the following erratum to this paper [1]:

The addition of authors Youri C. B. Broekhuizen and Monique W. M. Jaspers due to an omission. Additionally, their affiliation and emails: Center of Human Factors Engineering of Health Information Technology (HIT-Lab), Institute of Biomedical Informatics, Amsterdam Public Health Research Institute—Location Academic Medical Center (AMC), University of Amsterdam (UvA), 1012 WX Amsterdam, The Netherlands; youribroekhuizen@me.com (Y.C.B.B.); m.w.jaspers@amc.uva.nl (M.W.M.J.); their contributions: writing—original draft: Y.C.B.B.; conceptualization, writing—review and editing: M.W.M.J.

The authors would like to apologize for any inconvenience caused to the readers by these changes.
